# The fusiform gyrus exhibits an epigenetic signature for Alzheimer’s disease

**DOI:** 10.1186/s13148-020-00916-3

**Published:** 2020-08-27

**Authors:** Dingailu Ma, Irfete S. Fetahu, Mei Wang, Rui Fang, Jiahui Li, Hang Liu, Tobin Gramyk, Isabella Iwanicki, Sophie Gu, Winnie Xu, Li Tan, Feizhen Wu, Yujiang G. Shi

**Affiliations:** 1 Laboratory of Epigenetics, Institutes of Biomedical Sciences, Fudan University, Shanghai, 200032 China; 2 Key Laboratory of Birth Defects, Children’s Hospital of Fudan University, Shanghai, 201102 China; 3 Division of Endocrinology, Diabetes and Hypertension, Department of Medicine, Brigham and Women’s Hospital, Harvard Medical School, Boston, MA 02115 USA; 4 Department of Geriatrics, Shanghai General Hospital, Shanghai, 200080 China

**Keywords:** Alzheimer’s disease, Fusiform gyrus, Base-resolution DNA methylome analysis, Genome-wide transcriptome analysis, Protein-protein interaction networks, Postmortem brain, iPSC-derived neuron, Epigenetics

## Abstract

**Background:**

Alzheimer’s disease (AD) is the most common type of dementia, and patients with advanced AD frequently lose the ability to identify family members. The fusiform gyrus (FUS) of the brain is critical in facial recognition. However, AD etiology in the FUS of AD patients is poorly understood. New analytical strategies are needed to reveal the genetic and epigenetic basis of AD in FUS.

**Results:**

A complex of new analytical paradigms that integrates an array of transcriptomes and methylomes of normal controls, AD patients, and “AD-in-dish” models were used to identify genetic and epigenetic signatures of AD in FUS. Here we identified changes in gene expression that are specific to the FUS in brains of AD patients. These changes are closely linked to key genes in the AD network. Profiling of the methylome (5mC/5hmC/5fC/5caC) at base resolution identified 5 signature genes (*COL2A1*, *CAPN3*, *COL14A1*, *STAT5A*, *SPOCK3*) that exhibit perturbed expression, specifically in the FUS and display altered DNA methylome profiles that are common across AD-associated brain regions. Moreover, we demonstrate proof-of-principle that AD-associated methylome changes in these genes effectively predict the disease prognosis with enhanced sensitivity compared to presently used clinical criteria.

**Conclusions:**

This study identified a set of previously unexplored FUS-specific AD genes and their epigenetic characteristics, which may provide new insights into the molecular pathology of AD, attributing the genetic and epigenetic basis of FUS to AD development.

## Background

Alzheimer’s disease (AD) is a progressive neurodegenerative disorder with no cure or reliable methods for early detection [1–4]. Genetic studies show that early-onset AD (EOAD) (< 65 years old), accounting for only 5% of the AD population, is associated with mutations in the *APP*, *PSEN1*, and *PSEN2* genes. Meanwhile, ~ 50% of late-onset AD (LOAD) cases are attributed to homozygous *APOE4* [5]. However, the majority of AD cases are sporadic and cannot be explained by genetic variations, suggesting the existence of yet unknown etiology [5].

The symptoms and severity of AD vary in patients [6–8], which may be associated with the affected brain areas and lesion invasion rate. The fusiform gyrus (FUS) is a structure that lies on the basal surface of the temporal and occipital lobes in Brodmann’s area 37. It contains the critical fusiform face area (FFA) responsible for facial recognition [9]. Patients with advanced AD frequently lose the ability to identify family members. Likewise, subjects with mild cognitive impairment (MCI), who experience a higher risk of conversion to AD, possess distinct changes in functional connectivity of the FUS [10]. Therefore, AD-linked genes in the FUS may be critical in AD onset and progression and are thus promising targets for early diagnosis and therapy. However, compared with well-studied and documented brain areas such as the hippocampus (HPC) [11], prefrontal cortex (PFC) [12], and temporal lobe (TPL) [13], the gene expression characteristics and molecular mechanisms of action involved in AD pathology in the FUS [14] remain unexplored. The identification of AD-related expression and epigenetic signatures in different brain regions can provide support of inherent molecular mechanisms for the heterogeneous symptoms and improve the individualized early detection, prevention, and treatment of AD patients [15].

Growing lines of evidence suggest that epigenetic mechanisms play a crucial role in AD onset and development [13, 16, 17]. DNA methylation at the 5th position of cytosine (5mC) can be oxidized into 5-hydroxymethylcytosine (5hmC), 5-formylcytosine (5fC), and 5-carboxylcytosine (5caC) (hereafter referred to as 5mC, 5hmC, 5fC, and 5caC) by the family of ten-eleven translocation enzymes (*TET1*/*TET2*/*TET3*) in a stepwise manner [18]. Previous studies have indicated that AD onset and progression are linked to specific changes in DNA methylation in affected brain regions [19–21]. Our recent genome-wide DNA methylome analysis of postmortem brains and iPSC-derived neurons at base resolution identified a roadmap of AD-specific epigenetic signatures [17]. Moreover, the DNA methylome can be changed before the accumulation of pathological lesions and clinical manifestation [22–27]. In-depth study of mechanisms of gene expression and DNA methylation regulation in different brain regions of AD have shed new light on molecular markers of AD. Once verified in future, these specific sets of markers will have valuable implications for the early diagnosis of AD.

In this study, using a new analytical strategy, we uncovered an unprecedented FUS-specific AD gene expression profile and described an epigenetic basis for how AD-related changes extend to other brain regions beyond the FUS. Using independent methylation datasets from AD patients, we identified 5 genes (*COL2A1*, *CAPN3*, *COL14A1*, *STAT5A*, and *SPOCK3*) with a methylome signature that was significantly associated with AD prognosis.

## Results

### Changes in AD-specific gene networks are linked to specific brain regions

**Fig. 1:**
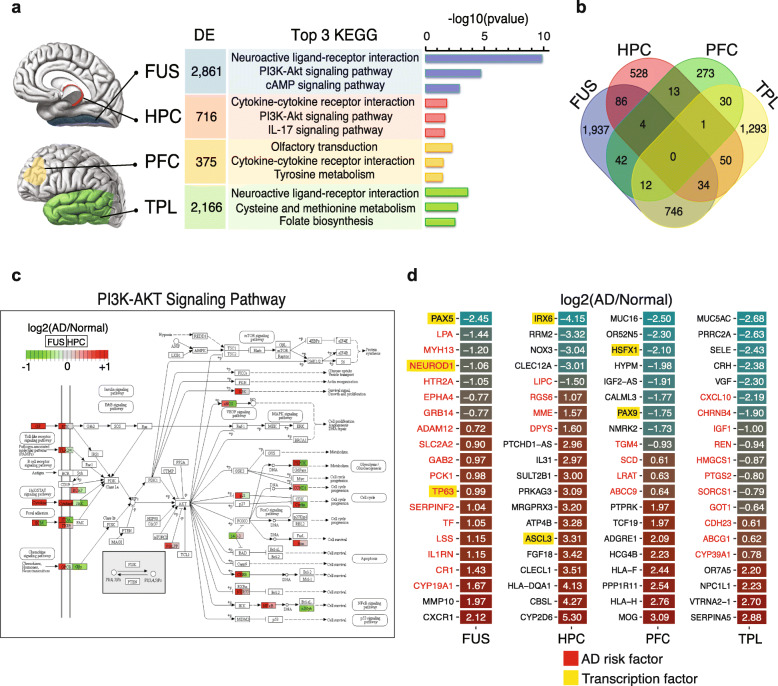
Differential gene expression analyses of four brain regions. **a** Illustration of the 4 studied brain areas, the numbers of differentially expressed (DE) genes in each region (fold-change > 1.5, *P* value < 0.05), and the top 3 involved KEGG pathways for each group of DE genes. Brain anatomy figures were modified from cases provided courtesy of A.Prof Frank Gaillard (Radiopaedia.org, rID: 47208, 46670). **b** Venn diagrams showing the overlap of the DE genes in **a**. **c** PathView plot shows the PI3K-AKT signaling pathway (KEGG: hsa04151) and relevant genes involved in the FUS (left of gene-box) and HPC (right of gene-box). Color key represents log2 (fold-change) of expression in AD compared to normal controls. **d** Heatmaps show log2 (fold-changes) of representative brain-region-specific DE genes. The absolute log2 (fold-changes) were sorted from high to low. The top 20 genes were selected. The number in each box is the log2 (fold-change)

By comparing transcriptomes of AD patients with normal controls (*n* = 108) in 4 different brain regions, we identified 2861 differentially expressed (DE) genes in the fusiform gyrus (FUS), 716 in the hippocampus (HPC), 375 in the prefrontal cortex (PFC), and 2166 in the temporal lobe (TPL) (Fig. 1a, Table S1). The top 3 enriched Kyoto Encyclopedia of Genes and Genomes (KEGG) pathways associated with the DE genes were distinct across brain regions, while sharing some similarities (Fig. 1a). DE genes in the HPC, the first affected area during AD onset, were enriched in cytokine-cytokine receptor interactions and the IL-17 signaling pathway, which are involved in acute and chronic inflammatory responses. In contrast, in the FUS, which is presumed to be a later-affected area in AD progression, the top-enriched pathways were neuroactive ligand-receptor interaction and the cAMP signaling pathway. Furthermore, DE gene overlap analysis among the four brain regions revealed that very few genes were consistently differentially expressed in more than two brain regions, and most genes were specifically affected in one unique brain region (Fig. 1b). We should note that comparing the DE analysis between brain regions only in controls, we found 4315 overlapping DE genes were differentially expressed in FUS, while there were 865, 1993, and 1512 overlapping DE genes in HPC, TPL, and PFC, respectively (Figure S1a-d). The apparent difference between DE genes among brain regions only in control and in AD samples suggests the unique gene expression regulation pattern in the context of AD.

Some of the KEGG pathways were significantly enriched in more than one brain region. We found that DE genes in both the FUS and TPL were significantly associated with neuroactive ligand-receptor interactions, while the HPC and PFC DE genes were associated with cytokine-cytokine receptor interaction. In addition, DE genes in the FUS and HPC were closely associated with the PI3K-Akt signaling pathway. Intriguingly, although DE genes in both the FUS and HPC were enriched in the PI3K/AKT/GSK-3β pathway, which is likely activated by neurotrophins and plays critical roles in AD onset and progression [28, 29], 84% (21 of 25) of the DE genes associated with this pathway in the FUS were upregulated in AD patients compared to controls (Fig. 1c). In contrast, the majority of these genes were downregulated or unchanged in the HPC. These gene expression patterns were also found in pathways related to neuroactive ligand-receptor interactions and cytokine-cytokine receptor interactions (Figure S1e-f). For example, we observed downregulation of GABA receptor genes in the FUS and TPL of AD brains (labeled with an arrow in Figure S1a). Interestingly, it was recently reported that secreted APP may modulate synaptic transmission via GABA receptors. Downregulation of the GABA receptor gene in the FUS and TPL is possibly linked to aberrant synaptic transmission in AD-affected neurons [30]. Together, these findings strongly suggest that the gene expression pattern in these specific cellular pathways, while similarly affected, may act differently, or perhaps even in opposite, in different brain regions during AD progression.

Critically, through AD-linkage analysis of the top 20 DE genes specific to each region, identified based on an absolute log2 (fold-change) (Fig. 1d), we found that 45% (36/80) of these DE genes have been previously reported as AD risk factors (Table S2). The definition of the list of AD risk factors was composed by taking into account the genetic testing or variation resources. Of these DE genes, 17/20 were associated with the FUS, 4/20 were associated with the HPC and PFC, and 11/20 were associated with the TPL. In addition, in order to ensure that the genes did appear in the AD GWAS results, we carefully compared the AD genetic datasets of the International Genomics of Alzheimer's Project (IGAP) [31] and ALZGENE [32]. 47.5% (38/80) of the top 20 DEGs were identified in AD GWAS results (Table S2). We also performed the gene enrichment analysis using the hypergeometric distribution for MalaCards and IGAP, respectively, resulting in significant *P* values (*P*_MalaCards_ = 0.00061, *P*_IGAP_ = 0.03764). Taken together, these data suggest that specific genetic components are involved in different brain regions linked to AD onset and progression.

### iPSC-derived neurons recapitulate the transcription profiles of the fusiform gyrus

**Fig. 2:**
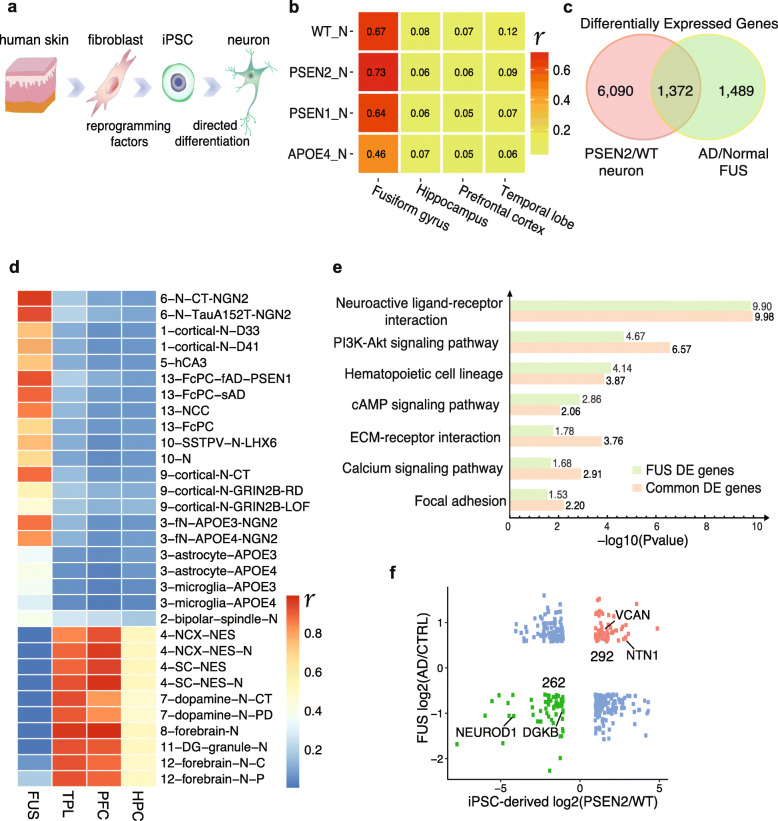
iPSC-derived neurons recapitulate the transcription profiles of the fusiform gyrus. **a** Schematic diagrams show the iPS cell differentiation process. **b** Heatmap shows expression correlation between iPSC-derived neurons and the 4 brain regions. Color scale represents Pearson’s correlation coefficient (*r*). *P* values in each cell line were < 0.01. Genes with FPKM > 1 were used to analyze correlation. WT_N: WT neuron, PSEN1_N: PSEN1^mut^ neuron, PSEN2_N: PSEN2^mut^ neuron, APOE4_N: APOE^ε4/ε4^ neuron. **c** Venn diagram of DE genes in PSEN2-N/WT-N at cutoff of fold-change > 2 and AD-FUS/normal at both fold-change > 1.5 and *P* value < 0.05. **d** Heatmap shows expression correlation between iPSC-derived neurons and 4 brain regions. Color scale represents Pearson’s correlation coefficient (*r*). *P* values in each cell line were < 0.01. Genes with FPKM > 1 were used to analyze correlation. Each row represents neurons derived from iPSCs using various differentiation protocols listed in Table S5. **e** KEGG enrichment comparison of the 1372 common DE genes and the FUS-specific DE genes. The number at the right of each bar is the corresponding −log10 (*P* value). **f** Scatter plot of log2 (fold-change) of the 1372 commonly DE genes in **c**. Representative AD risk factors are labeled

We sought to further characterize possible molecular changes relating to the region-specific genetic changes found in AD patients. Induced pluripotent stem cells (iPSCs) derived from AD patients allowed us to accurately recapitulate neurogenesis, providing suitable models to study AD [33]. We employed four types of iPSCs: WT, PSEN1^mut^, PSEN2^mut^, and APOE^ε4/ε4^, derived from normal individuals and AD patients carrying a *PSEN1* mutation, *PSEN2* mutation, and homozygous *APOE-ε4*, respectively. These iPSCs were then subjected to directed differentiation into neurons using a commonly available protocol [17, 34] (Fig. 2a). Unexpectedly, bioinformatic analysis showed the transcriptome profiles of WT neurons (WT-N), APOE4-N, PSEN1-N, and PSEN2-N were all strongly correlated with those of the FUS, with higher correlation coefficients than those of the HPC, PFC, and TPL (Fig. 2b). This indicated that the transcriptional features of iPSC-derived neurons were more similar to the FUS than other brain regions. PSEN2-N showed the strongest correlation with the FUS (*r* = 0.73, *P* value < 0.01, Pearson’s correlation), suggesting that these AD neurons, particularly PSEN2-N, may recapitulate the transcription profiles of AD in the FUS.

The similarity of the transcriptional profiles between the FUS and iPSC-derived cortical neurons may be due to the specific differentiation protocol used. To systematically evaluate the protocol bias-resultant similarity, we analyzed 13 up-to-date available public datasets of differentiated brain cell transcriptomes [34–48] (detailed information is summarized in Table S5) and surprisingly found that iPSC-derived cortical neurons generated following the same protocol [34] also had a stronger correlation with the FUS than other brain regions (Fig. 2d). In contrast, neurons differentiated through different protocols, such as DG-granule, forebrain, and dopamine neurons (last 6 rows in Fig. 2d), were more transcriptionally similar to the HPC, PFC, and TPL. These data again suggest that iPSC-derived cortical neurons possess similar transcriptional patterns to the FUS.

Next, we asked how many DE genes were shared between iPSC-derived AD neurons and the FUS when compared to their respective controls. Approximately 50% of DE genes in the FUS overlapped with those in iPSC-derived cortical neurons (Fig. 2c, Figure S2a-b). There were 1372 DE genes shared between the FUS and PSEN2-N (Fig. 2c), 1388 between the FUS and PSEN1-N, and 1475 between the FUS and APOE4-N (Figure S2a-b, Table S3). We also compared the overlap of DE genes between all cell lines tested: PSEN1-N, PSEN2-N, and APOE4-N (Figure S2c). Gene ontology analysis showed that the 1372 common DE genes between the FUS and PSEN2-N were significantly enriched in extracellular matrix organization, chemical synaptic transmission, cell differentiation, and axon genesis (Figure S2d). Importantly, the top-enriched KEGG pathways in these 1372 common DE genes were also significantly enriched in the FUS DE genes (Fig. 2e). There are 292 genes that are upregulated and 262 genes that are downregulated in both the FUS and PSEN2-N (Fig. 2f, Table S4). Interestingly, these 554 (292 + 262) DE genes were distributed in extracellular and membrane areas in the PI3K/AKT/GSK-3β pathway (Figure S2e), implying a relationship to signal transduction and neuronal cell survival [49]. Collectively, iPSC-derived neurons recapitulate the transcription profiles of the diseased FUS, suggesting that the molecular pathology of AD in PSEN2-N significantly mirrors that in the FUS.

### Protein-protein interaction networks reveal FUS-specific key AD-associated genes

**Fig. 3:**
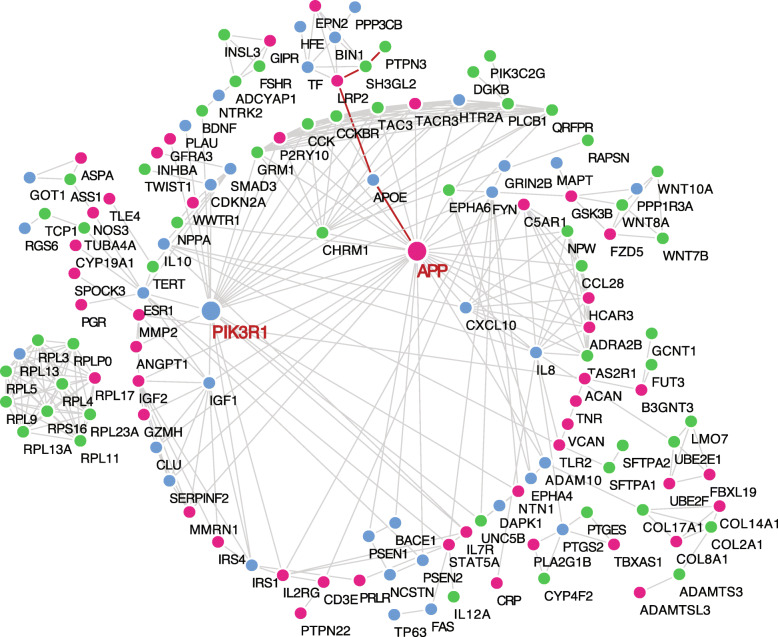
Protein-protein interaction networks reveal FUS-specific key AD-associated genes. Protein-protein interactions (PPI) between known AD risk factors and 554 shared DE genes in PSEN2-N and AD-FUS. Blue dots represent known AD risk factors, red and green dots stand for up- and downregulated DE genes in AD, respectively

To determine if these 554 DE genes shared by the FUS and PSEN2-N have a clear association with well-defined AD genes, we used the STRING [50] analytical tool to reveal the protein-protein interaction (PPI) networks (Fig. 3). Only interaction edges with a high confidence (interaction score ≥ 0.9, PPI enrichment *P* value < 1e−11) were selected. STRING analysis identified 114 DE genes as being clustered to well-defined key AD genes in a hierarchical fashion (Table S6). For example, with the ε4 allele being an AD risk factor [51], *APOE* was the primary nodule connecting to *APP* (red line), which encodes peptides that form amyloid plaques in AD brains (Fig. 3). The secondary nodule linked to *APP* is the highly expressed *LRP2*, in which a SNP associated with AD susceptibility was found [52]. The nodule downstream of *LRP2* is *SH3GL2*, which is implicated in synaptic vesicle endocytosis and AD protein homeostasis [53]. The last nodule is *PTPN3*, which is a diabetes-related gene [54]. These connections imply that *LRP2*, *SH3GL2*, and *PTPN3* are possible novel AD-linked genes which play critical roles that are closely associated with the *APOE4* functional network in AD pathology. Using the same approach, we performed PPI enrichment for PSEN1-N and APOE4-N and identified 112 and 116 enriched DE genes associated with the functional realm of the other well-defined key AD genes, respectively (Figure S3-4, Table S6).

We found common and unique sub-networks among these PPI networks. The *APP*-centered sub-network appeared in each network (Fig. 3, Figure S3-4), indicating that *APP* plays a critical role in AD progression and that the DE genes associated with the *APP* functional nodule may be deeply involved in amyloid plaque formation. Compared to APOE4-N, PSEN1-N and PSEN2-N had a unique *PIK3R1-*centered sub-network (Fig. 3 and Figure S3). *PIK3R1* is implicated in the metabolic actions of insulin, in which insulin receptor-activated genes play critical roles in vesicle transport and RNA splicing in neuronal cells [55, 56]. Interestingly, the APOE4-N PPI network had distinct patterns. The *NMU*-centered sub-network is connected to *APP* and was not observed in PSEN1-N and PSEN2-N (Figure S4). *NMU* encodes a member of the neuromedin family of neuropeptides, which play important roles in inflammation-mediated memory impairment and neuronal cell-death [57]. Taken together, these data revealed that the AD-specific gene expression signature shared by the FUS and in vitro differentiated cortical neurons is functionally linked to well-defined AD risk factors.

### AD-specific methylation patterns in the newly identified AD-specific gene expression signatures

**Fig. 4:**
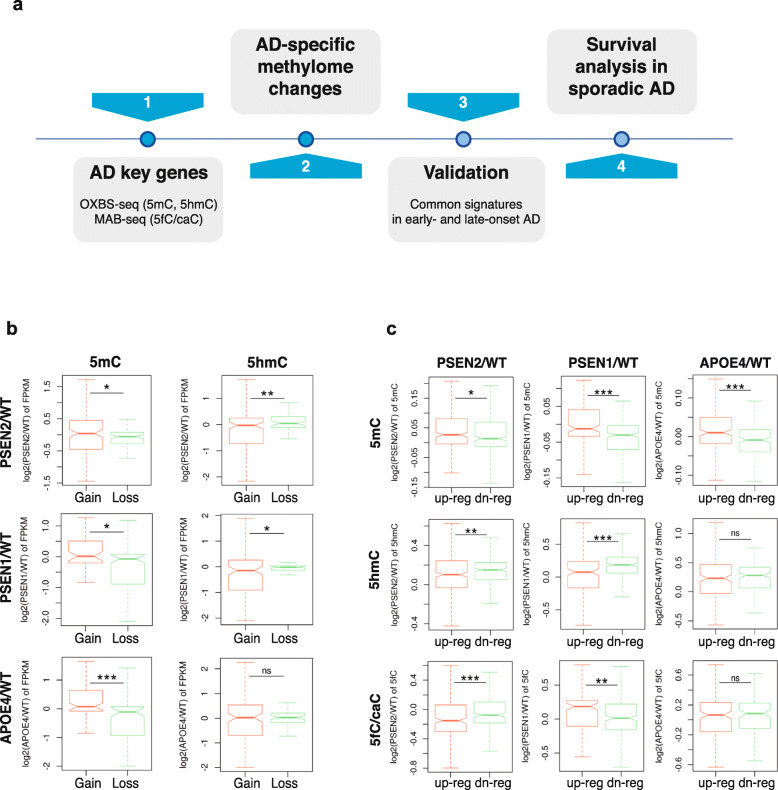
AD-specific methylation patterns in the newly identified AD-specific gene expression signatures. **a** Schematic diagram of the analysis workflow to identify AD-specific methylome patterns and AD-methylome signatures. **b**, **c** Boxplots show expression fold-changes in methylation-gain or loss on gene bodies of PPI-enriched key AD-associated genes (**b**), and 5mC/5hmC/5fC/caC fold-changes on the gene bodies of up- and downregulated DE genes (**c**). *P* values were calculated by the Wilcoxon rank-sum test (*P* < 0.05 (*); *P* < 0.01 (**); *P* < 0.001 (***); ns, not significant)

We identified AD-specific methylome patterns and signatures in the newly identified AD-specific gene expression signatures (Fig. 4a). Using oxBS- and MAB-seq, we profiled whole-genome 5mC, 5hmC, and 5fC/caC signals at base resolution in WT-N, PSEN1-N, PSEN2-N, and APOE4-N (Figure S5a-f). The global methylation patterns in the gene bodies varied between AD and WT iPSC-derived neurons, suggesting that aberrant DNA methylomes may contribute to transcriptional regulation in AD progression.

Next, we examined the correlation between the AD-specific gene expression signatures (Table S6) and the methylome. Genes were separated into two groups, gain or loss of methylation, according to the average methylation levels on their gene bodies, and then stratified by their expression fold-changes (Fig. 4b). Compared to all Refseq genes (Figure S5g), we observed unusual perturbation of the DNA methylomes in the AD-specific expression signature genes. Consistently in PSEN2, PSEN1, and APOE4 neurons, loss of 5mC on the gene bodies of the signature genes correlated with lower expression in AD neurons compared to WT (Fig. 4b, left panel). On the contrary, the genes with a loss of 5hmC showed higher expression in AD neurons than those with a gain of 5hmC (Fig. 4b, right panel).

We next investigated the gene body methylation differences in the up- or downregulated signature genes (Fig. 4c). The upregulated genes tended to gain more 5mC on their gene bodies than the downregulated genes in PSEN2-N compared to WT-N (Fig. 4c, top panel). This trend was also found in PSEN1 and APOE4 neurons (Fig. 4c, top panel). In contrast, downregulated genes tended to gain more 5hmC than the upregulated genes. Collectively, AD-specific methylome changes are significantly correlated with perturbed expression of the newly identified AD-specific signature genes.

### Cross-validation of key AD genes in independent methylation datasets

**Fig. 5:**
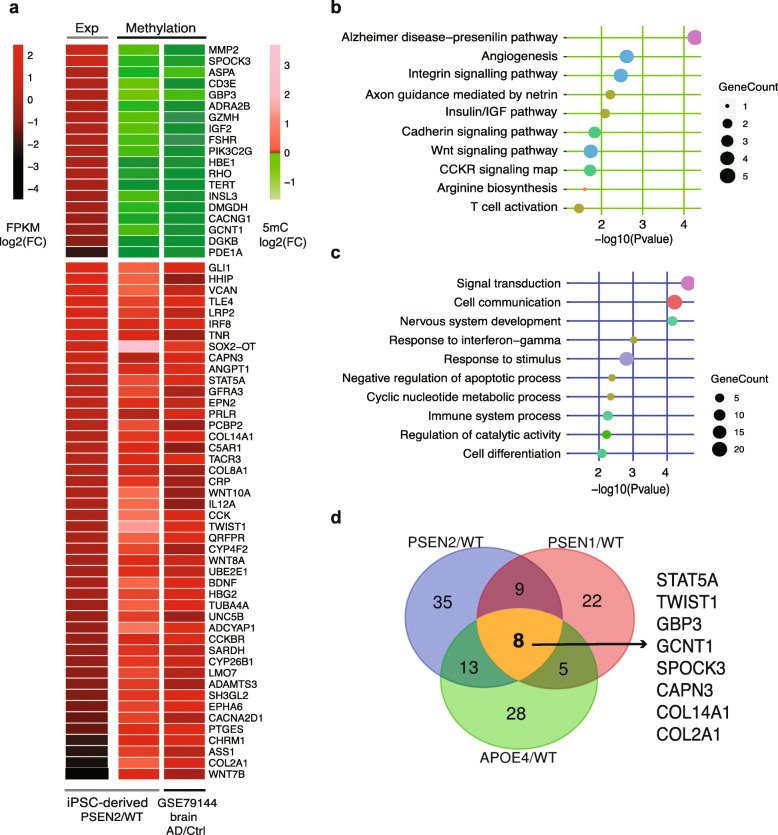
Cross-validation of key AD genes in independent methylation datasets. **a** Heatmaps show expression fold-changes and methylation fold-changes in PSEN2/WT. The methylation data are from GSE79144 (*n* = 44). FC, fold-change. **b**, **c** PANTHER pathway (**b**) and GO term (**c**) enrichment analyses for genes identified from PSEN2/WT neurons and validated in the GSE79144 dataset. **d** Venn diagrams show the overlap of genes identified from PSEN2/WT, PSEN1/WT, and APOE4/WT neurons and validated in the GSE79144 dataset with a similar methylation change trend

To validate that the identified methylation changes existed in the brain tissues of AD patients, we examined an independent cohort of 44 controls and AD patients and used the methylation data of the temporal cortex to verify the methylation sites we identified [58]. Our results showed that 65/114 (57%) of the newly identified signature genes in PSEN2-N had a consistent trend of methylation changes (Fig. 5a). Importantly, the top-enriched pathways or Gene Ontology (GO) terms associated with these 65 validated signature genes suggested critical roles in AD development, including the AD-presenilin pathway, axon guidance mediated by netrin, and nervous system development (Fig. 5b, c). Additionally, 44/112 (39%) enriched DE genes in PSEN1-N (Figure S6a) and 54/116 (47%) enriched DE genes in APOE4-N (Figure S6b) were validated to show a consistent trend of methylation change. Furthermore, among the validated genes, 38/65 (58%) in PSEN2-N, 27/44 (61%) in PSEN1-N, and 25/54 (46%) in APOE4-N were previously reported as being closely associated with AD (Table S7). Among all of these validated genes, 8 genes (*STAT5A*, *TWIST1*, *GBP3*, *GCNT1*, *SPOCK3*, *CAPN3*, *COL14A1*, and *COL2A1*) were altered in neurons derived from patients with PSEN1, PSEN2, and APOE4 mutations (Fig. 5d, Table S7). Intriguingly, there were 9 genes (*GLI1*, *CD3E*, *CRP*, *ANGPT1*, *ASS1*, *IGF2*, *INSL3*, *PDE1A*, and *PIK3C2G*) that were altered only in PSEN1 and PSEN2 neurons, suggesting they might be related to EOAD-specific signatures.

Among the 8 validated common signature genes, two are transcription factors (*STAT5A*, *TWIST1*), two are associated with immune response (*GBP3*, *GCNT1*), one is a nervous system regulator (*SPOCK3*), and three are intra- or extracellular components (*CAPN3*, *COL2A1*, *COL14A1*) (Table S7). These genes are directly or indirectly associated with AD pathophysiology. For example, the JAK2-STAT5 signaling pathway plays a critical role in mediating IL-3-induced activation of microglia during AD pathogenesis [59]. *TWIST1* is targeted by an AD-specific miRNA in a set of AD patients and is an important upstream mediator of mutant Htt (huntingtin protein)-induced neuronal death [60]. *COL14A1* is an interactive gene in the gut-brain axis in AD [61]. Collectively, these results indicate that the validated signature genes likely play important roles in AD progression and suggest that accumulation of aberrant methylomes, which may predate abnormal transcriptional changes, may function as a new set of independent epigenetic markers for early detection and prognostic evaluation of AD.

### Survival analysis of validated AD signatures using the Religious Orders Study and Rush Memory and Aging Project (ROS/MAP) cohort

**Fig. 6:**
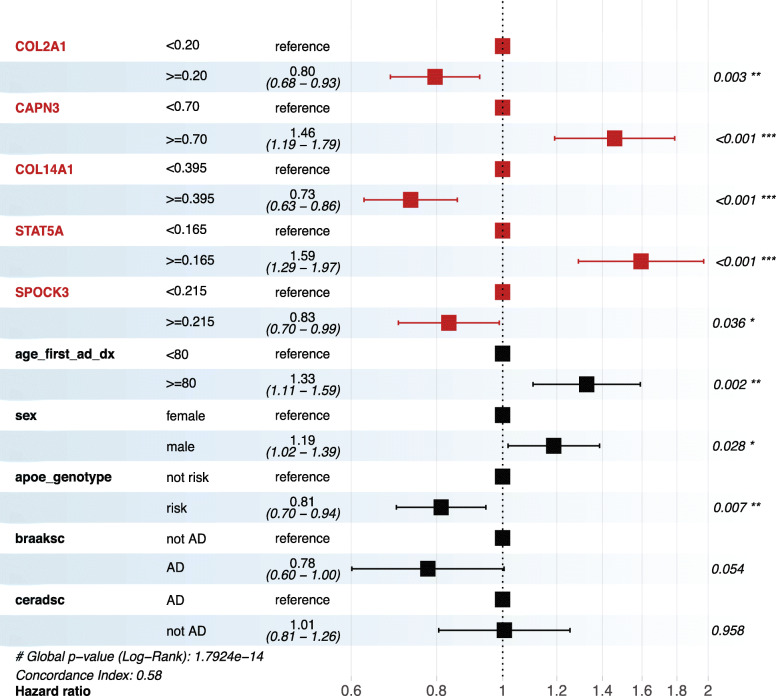
Survival analysis of validated AD signatures with the ROS/MAP cohort. Forest plots show the hazard ratios (HR) of identified 5mC signatures and clinical features derived from Cox proportional hazards models in the ROS/MAP cohort (*n* = 174). HR > 1 indicates an increased risk of death, while HR < 1 indicates a decreased risk. *P* values were calculated by the log-rank test

To investigate whether the 8 commonly validated genes are associated with the prognosis of AD, we evaluated the effect of changes in the methylome on these potential risk factors on the survival time of AD patients. A total of 174 AD patients in the ROS/MAP cohort [62] were included with censored follow-up time from the first diagnosis to the age of death. Other clinical characteristics from medical records including sex, *APOE* genotype, BRAAK-score, and CERAD-score were included. Among the 8 genes, the methylation levels of 5 gene signatures (*COL2A1*, *CAPN3*, *COL14A1*, *STAT5A*, and *SPOCK3*) were detected in the ROS/MAP cohort. The Cox proportional hazards regression model was used to find risk factors for AD patients’ deaths (Fig. 6). Higher methylation levels on the gene bodies of *CAPN3* (HR = 1.46, CI = 1.19–1.79, *P* < 0.001) and *STAT5A* (HR = 1.59, CI = 1.29–1.97, *P* < 0.001) were associated with significantly increased risk of death in AD patients, while higher methylation levels on *COL2A1* (HR = 0.80, CI = 0.68–0.93, *P* = 0.003), *COL14A1* (HR = 0.73, CI = 0.63–0.86, *P* < 0.001), and *SPOCK3* (HR = 0.83, CI = 0.70–0.99, *P* = 0.036) were associated with significantly decreased risk of death. Apart from the AD-specific methylome changes, patients have lower death risk if they are younger when first diagnosed with AD [63]. Peculiarly, male AD patients seem to have greater risk of death [63]. Although BRAAK and CERAD scores are important AD diagnosis criteria, they are barely correlated with AD prognosis. Together, these data suggest that the methylome signatures are more sensitive than traditional clinical markers in determining AD prognosis.

## Discussion

Amyloid deposits and neurofibrillary tangles are well-characterized and common pathological features of AD brains. The aging-related temporal sequence of how these pathological features unfold is well-documented and studied. However, the spatial/regional events of AD pathogenesis and progression are currently poorly understood, although it has been long noticed that disparities in symptoms and severity of AD progression are specifically associated with neurodegeneration and disordered neurogenesis in different brain regions [64]. Using the experimental strategy outlined in the Graphic Abstract, we are just beginning to understand how different cellular and molecular pathways in different brain regions are linked and contribute to AD onset and progression.

In the present study, we initially analyzed the transcriptomes of 4 dissected brain sub-regions (FUS, HPC, PFC, and TPL) of both AD patients and normal controls. These analyses revealed novel and distinct gene expression signatures in the different brain areas of AD patients. For example, NEUROD1, a well-defined AD risk factor activated by Wnt signaling, which promotes adult neuron maturation [65], was ranked among the top DE genes of the FUS. Concurrently, cytokine-cytokine receptor interaction was the top perturbed pathway in both the HPC and PFC, implying that the immune response is potentially impaired in these areas in AD patients. These findings suggest that there are distinct molecular and cellular mechanisms by which a spatial-specific occurrence of neurodegeneration and impairment of neurogenesis in different brain regions is independently triggered or coordinated to contribute to AD symptoms.

Selective neuronal loss in vulnerable brain regions is the neuropathological hallmark of AD. Currently, the epigenetic mechanisms of AD neuronal loss are poorly understood and studied. Functional analysis of the differentially methylated genes in AD showed that a large part of them are associated with neurodevelopment and neurogenesis, indicating that the abnormal DNA methylome in AD may break the normal functional balance in the process of neurodevelopment and neurogenesis, causing potential neuronal loss [66]. By further determining those differentially methylated sites and regions, we found that they overlapped with vital regulatory elements in the genome, such as CpG islands, poised promoters with bivalent histone marks, and enhancers [19, 27, 66]. Enhancer hypomethylation in AD neurons could significantly affect the expression of genes involved in neurogenesis pathways [27]. Poised promoters are essential for neural development and maintenance of lineage differentiation [67]. We speculate that, under the influence of AD, abnormal fluctuations in DNA methylation levels occur on important genomic regulatory elements such as promoters and enhancers, resulting in aberrant expression of genes related to neurodevelopment and neurogenesis. In addition, there are evident epigenetic and transcriptional losses in cell cycle control in AD neurons [27]. For example, hypomethylation of enhancers in AD neurons may upregulate cell cycle genes and promote neuronal death and synapse loss. Therefore, it is possible that hypomethylation of enhancers that affect neurogenesis genes may be the molecular basis and initial cause for a close relationship between the burden of neurofibrillary tangles and neuronal loss in AD [27].

We discovered that the gene expression signature of AD in the FUS had the closest similarity to that of in vitro iPSC-derived AD neurons. STRING analysis of the FUS/iPSC-N commonly shared gene expression signature identified a set of genes specifically linked to well-documented key AD genes such as *APP* and *APOE*. iPSC-derived neurons that resemble the fusiform gyrus in their transcription profiles were generated using a specific and common protocol [34]. Our transcriptional correlation analysis further indicated that the similarity of gene expression signatures between the iPSC-derived neurons and distinct brain regions depends on the differentiation protocols, and possibly the involved growth factors (e.g., *NGN2* and *GRIN2B*). The exact reasons for this are currently unknown and warrant future investigation. The discovery that the iPSC-derived neurons in our study [34] shared a gene expression signature with the FUS provides an excellent and unique tool to study the molecular pathology and mechanisms of action in the FUS during AD onset and progression. These findings also remind us to re-think the in vitro neuron differentiation process. In particular, extra caution will need to be taken to ensure proper application of a specific neuronal differentiation protocol when establishing models to study neurodegenerative diseases.

We should note that there are several challenges in comparing in vitro data from homogeneous cultures of iPSC-derived neurons with data obtained from highly heterogeneous brain tissues [68–74]. First, the composition of cells in brain tissue is complex. The correlation of expression profiles between FUS and iPSC neurons is likely to be affected by other cell types, such as glia. Second, when iPSCs are reprogrammed, it is necessary to select appropriate clones to continue the cultivation. This process is not only difficult to standardize, but also merely reflects the nature of a single cell colony. Third, compared with the original neurons of the patient, neurons differentiated in vitro lack the connection and interaction between different brain cells. In future investigations, it is important and necessary to map the cell type- and brain region-specific transcriptional and epigenetic landscapes to disease phenotypes [72]. Nonetheless, despite these potential limitations, it has been reported that transcriptional signatures of schizophrenia in iPSC-derived NPCs and neurons are concordant with post-mortem brains [75]. We are confident that the similarity of gene expression signatures between iPSC-neurons and FUS observed in both normal controls and AD may provide new clues to the molecular mechanisms of AD in FUS.

Genetic and epigenetic risk factors can independently affect the same diseased gene [76]. It is possible that the DNA methylome alterations might be the functional consequence of genetic variants associated with disease susceptibility. On the other hand, epigenetic factors, such as adverse environmental cues or aging, may directly reprogram the epigenome, which, in turn, may alter the expression-associated genes to result in neurodegenerative diseases [76]. In our previous study [17], we reported the first genome-wide roadmap of epigenetic signatures in AD based on the methylated DNA base cytosine [17]. Significantly, in the present study, we determined novel FUS-specific AD genes, whose transcriptional alterations were significantly linked to key AD risk factors. We identified five AD signature genes with a methylome signature that was significantly associated with AD prognosis in ROS/MAP cohorts. Moreover, the methylome signature is not only restrained to the FUS, but is also commonly shared among other brain regions. We envision that these epigenetic signatures are more generalized and likely to be epigenetic codes for AD rather than simply the consequence of AD progression.

## Conclusions

Using a complex of new analytical paradigms that integrates transcriptomes and methylomes of normal controls, AD patients, and “AD-in-dish” models, we identified a set of previously unexplored FUS-specific AD genes (*COL2A1*, *CAPN3*, *COL14A1*, *STAT5A*, and *SPOCK3*) and their epigenetic characteristics, which may provide new insights into the molecular pathology of AD. Moreover, this study first reports the molecular link between FUS and AD, which uncovers the genetic/epigenetic basis of FUS contributing to the spatial/regional events of AD pathogenesis, leading to new insights into how molecular changes in different brain regions affect AD onset and progression. The FUS-specific genetic/epigenetic signatures may be potential biomarkers for AD etiology.

## Methods

### Cell lines

iPSCs and iPSC-derived cortical neurons were obtained from Axol Biosciences (Cambridge, UK). The PSEN1^mut^ cell line carries the *PSEN1* gene mutation L286V, and the PSEN2^mut^ cell line carries the *PSEN2* gene mutation N141I. Both types of mutations are genetic risk factors for familial AD. The APOE^ε4/ε4^ cell line carries a homozygote for the APOE ε4 allele, which is a genetic risk factor for sporadic AD. iPSCs were reprogrammed from skin fibroblasts of AD patients and normal controls. Skin fibroblasts used for reprogramming PSEN1^mut^ and PSEN2^mut^ cell lines were from 38-year-old female and 81-year-old male AD patients, respectively. Fibroblasts for reprogramming APOE^ε4/ε4^ cell line were from an 87-year-old female patient with sporadic AD. Directed differentiation of iPSCs to cortical neurons was performed as described previously [77].

### Oxidative and methylase-assisted bisulfite sequencing

Oxidative (oxBS-seq) and methylase-assisted bisulfite sequencing (MAB-seq) at single-base-pair resolution were performed as previously described [17]. We performed oxBS-seq and MAB-seq in all cell lines in the AD patient samples reported in this study. All libraries were sequenced using Illumina HiSeq X Ten platform, generating at least 100 GB of data for each sample, allowing for whole-genome methylation analysis at base resolution.

### Identification of 5mC, 5hmC, and 5fC/5caC sites

We evaluated the data quality of oxBS-seq and MAB-seq [17] and used the bsmap (v2.74) [78] software package to align the reads to the human reference genome of UCSC (hg19) and to identify the methylation signals. We retained CpG sites with a sequencing depth of at least 10× for downstream analysis. To accurately calculate 5mC and 5hmC signals, we used home-made R script to ensure that the chromosome coordinates of these CpG sites are consistent between each pair of BS and oxBS libraries. Then we used the “mlml” script in methpipe package to identify the 5mC and 5hmC signals at these CpG sites. To calculate 5fC/caC signals in the binomial distribution model, we used M.SssI enzyme and bisulfite conversion inefficiency of 1.64% (measured in preliminary experiments) to correct the 5fC/caC signals. We only retained 5fC/caC sites with sequencing depth ≥ 10, *P* value < 0.01, and FDR < 0.01 for downstream analysis. In addition, given that DNA samples may carry sequence mutations different from the reference genome, this may lead to false positives when calculating the 5mC, 5hmC, and 5fC/caC signals. We used Biscuit software package (https://github.com/zwdzwd/biscuit) to identify and remove these potential mutation sites with default parameters. The remaining loci are used for downstream analysis.

### Normalization of 5mC, 5hmC, and 5fC/5caC signals

In order to compare the 5mC, 5hmC, and 5fC/caC signals between different samples and exclude the effect of the difference in sequencing depth, we scanned the entire genome in non-overlapping 1000 bp bins to normalize the signals. The 5mC and 5hmC signals were represented by TNC / (TNC + TNT), where TNT and TNC, respectively, represent the total number of T and the total number of C in the region. The 5fC/caC signal was represented by the average value in this region, because the density of the 5fC/5caC sites in the genome is much lower than 5mC or 5hmC.

### mRNA-seq

The total mRNA was isolated using TRIzol according to the manufacturer’s instructions (Invitrogen, CA, USA). Libraries were generated and sequenced at WuxiNextCode (Shanghai, China). For each sample, > 40 million paired-end reads with Q30 > 90% were generated. Reads were then mapped onto the hg19 genome using TopHat (v2.1.1) [79]. Quantification of gene expression was performed using featureCounts in the Rsubread package (v1.32.4) [80]. RPKM of genes was calculated using the “rpkm” function in the edgeR package (v3.24.3) [81] and gene annotations were determined through the built-in annotation in the featureCounts package. Differentially expressed genes were identified using the edgeR [81] package with a cutoff RPKM-fold-change > 1.5 and *P* value < 0.05 in AD samples versus normal controls.

### Digital deconvolution of bulk tissues

Cell-type deconvolution was performed using CIBERSORTx (http://cibersortx.stanford.edu), which is an analytical tool developed by Newman et al. [27] to impute gene expression profiles and provide an estimation of the abundances of member cell types in a mixed cell population, using gene expression data. We used a gene signature matrix (involving 903 cell-specific marker genes) derived from single-cell RNA-seq measures in adult human brain cells (signature matrix [82]; source [83]). CIBERSORTx was run with batch correction and 100 permutations.

### Known AD risk factors

Known AD risk factors were obtained from the MalaCards-HUMAN DISEASE DATABASE (https://www.malacards.org/card/alzheimer_disease). Susceptible genes and risk factors were summarized according to the “Genes” section. The gene list used in our analysis was provided in Table S9.

International Genomics of Alzheimer’s Project (IGAP) is a large two-stage study based upon genome-wide association studies (GWAS) on individuals of European ancestry. In stage 1, IGAP used genotyped and imputed data on 7,055,881 single nucleotide polymorphisms (SNPs) to meta-analyze four previously-published GWAS datasets consisting of 17,008 Alzheimer’s disease cases and 37,154 controls (The European Alzheimer’s disease Initiative (EADI), the Alzheimer Disease Genetics Consortium (ADGC), The Cohorts for Heart and Aging Research in Genomic Epidemiology consortium (CHARGE), The Genetic and Environmental Risk in AD consortium (GERAD)). In stage 2, 11,632 SNPs were genotyped and tested for association in an independent set of 8572 Alzheimer’s disease cases and 11,312 controls. Finally, a meta-analysis was performed combining results from stages 1 and 2.

### Gene ontology (GO) and Kyoto Encyclopedia of Genes and Genomes (KEGG) enrichment analysis

GO and KEGG enrichment analyses were performed using the Database for Annotation, Visualization and Integrated Discovery (DAVID) website [84, 85]. Visualization of KEGG pathways was conducted using Pathview (v1.22.3) [86].

### Other statistical analyses

Continuous variables were descriptively summarized using medians with 25th and 75th percentiles, and categorical factors were reported using percentages. R package “VennDiagram” was used to determine the groupings of values that were presented in the Venn diagram. The Pearson correlation coefficient was calculated to measure the linear correlation of the gene expression between iPSC-derived neurons and the four brain regions (*P* values < 0.01). The STRING [50] analytical tool was used to reveal the protein-protein interaction (PPI) network, with only high-confidence interaction edges kept for downstream analyses (interaction score ≥ 0.9, PPI enrichment *P* value < 1e−11). Boxplots were used to describe the distribution and patterns of methylation changes in key AD-associated genes. The statistical significance of the methylation changes between different groups was determined by the Wilcoxon rank-sum test using R package “stats.” The multivariable Cox proportional hazards regression model was used to find risk factors for AD patients’ deaths. The Kaplan-Meier survival analysis was used to predict the survival probabilities at distinct methylation level cutoffs. *P* values were calculated by the log-rank test.

## Supplementary information

**Additional file 1: Figures S1-S6.** Supplementary_figures_merge.pdf.

**Additional file 2: Table S1.** ST1_DEG_of_4_brain_reigons.xls.

**Additional file 3: Table S2.** ST2_Top_DEG_in_heatmap_AD-risk-factor_TF.xlsx.

**Additional file 4: Table S3.** ST3_Venn_common_genes_in_FUS_and_AD-Ns.xlsx.

**Additional file 5: Table S4.** ST4_common_up_down_expressed_genes_in_FUS_and_AD-Ns.xlsx.

**Additional file 6: Table S5.** ST5_details_of_neurons_differentiation_protocols.xlsx.

**Additional file 7: Table S6.** ST6_STRING_output_interactions.xlsx.

**Additional file 8: Table S7.** ST7_validated_genes_function_annotation.xlsx.

**Additional file 9: Table S8.** ST8_Samples_info.xlsx.

**Additional file 10: Table S9.** ST9_Known_AD_risk_factors.txt. All computer codes used in our analyses were deposited in GitHub.

## Data Availability

Whole-genome sequencing data for 5mC, 5hmC, and 5fC/caC modifications have been deposited in the Sequence Read Archive (SRA) with accession code PRJNA476128, along with RNA sequencing data PRJNA557835, and are available from the authors upon request. All publicly available datasets were already cited when mentioned in the manuscript. The mRNA-seq datasets of different brain regions, including fusiform gyrus (GSE95587), hippocampus (GSE67333), prefrontal cortex (GSE53697), and temporal lobe (GSE104704) (Table S8), were obtained from the GEO website (https://www.ncbi.nlm.nih.gov/geo/). The accession numbers for mRNA-seq data of other iPSC-derived cell lines are GSE87963, GSE90469, GSE107514, GSE111977, GSE112732, GSE114685, GSE115205, GSE102352, GSE102956, GSE114553, GSE58933, GSE63734, and GSE104141 (Table S5). In order to verify the AD methylation signatures in other independent AD datasets, we analyzed the bisulfite-converted DNA data from 44 brain tissues, including 22 normal controls and 22 AD patients (Table S8) (GSE79144). In addition, in order to assess the potential significance of the AD methylation signatures in the prognosis of AD, we performed the analysis of death risks and survival probabilities with the ROS/MAP (Religious Orders Study and Rush Memory and Aging Project) cohort study dataset [62]. The ROS/MAP methylation dataset includes DNA methylation data from 708 subjects’ prefrontal cortex tissues.

## Abbreviations

AD: Alzheimer’s disease
FUS: Fusiform gyrus
HPC: Hippocampus
PFC: Prefrontal cortex
TPL: Temporal lobe
DEG: Differentially expressed genes
iPSC: Induced pluripotent stem cell
WT-N, PSEN1-N, PSEN2-N, APOE4-N: iPSC-differentiated neurons, derived from normal individuals and AD patients carrying a *PSEN1* mutation, *PSEN2* mutation, or homozygous *APOE-ε4*, respectively
5mC: 5-Methylcytosine
5hmC: 5-Hydroxymethylcytosine
5fC: 5-Formylcytosine
5caC: 5-Carboxylcytosine
PPI: Protein-protein interaction
ROS/MAP: The Religious Orders Study and Rush Memory and Aging Project cohorts
EOAD: Early-onset Alzheimer’s disease
LOAD: Late-onset Alzheimer’s disease
