# Supplementary material for: The fusiform gyrus exhibits an epigenetic signature for Alzheimer’s disease
Source: Clin Epigenetics. 2020 Aug 27;12:129. doi: 10.1186/s13148-020-00916-3 (PMC7457273; doi:10.1186/s13148-020-00916-3)

The fusiform gyrus exhibits an epigenetic signature for Alzheimer's disease

Supplementary figures

Dingailu Ma[1,2], Irfete S. Fetahu[2], Mei Wang[3], Rui Fang[2], Jiahui Li[1], Hang Liu[1], Tobin Gramyk[2], Isabella Iwanicki[2], Sophie Gu[2], Winnie Xu[1], Li Tan[1], Feizhen Wu[1,*], Yujiang G. Shi[2,*]

[1]Laboratory of Epigenetics, Institutes of Biomedical Sciences, Fudan University, Shanghai, 200032, China, and Key Laboratory of Birth Defects, Children's Hospital of Fudan University, Shanghai, 201102, China.

[2]Division of Endocrinology, Diabetes and Hypertension, Department of Medicine, Brigham and Women's Hospital, Harvard Medical School, Boston, MA, 02115, USA.

[3]Department of Geriatrics, Shanghai General Hospital, Shanghai, 200080, China.

*Corresponding author e-mail: yujiang_shi@hms.harvard.edu, wufz@fudan.edu.cn.

Supplementary figure legends

Figure S1.

(a-d) Venn diagrams showing the overlaps of the differentially expressed (DE) genes between brain regions only in controls. FUS (a): fusiform gyrus; HPC (b): hippocampus; TPL (c): temporal lobe; PFC (d): prefrontal cortex.

(e) PathView plot shows the neuroactive ligand-receptor interaction pathway (KEGG: hsa04080) and relevant genes involved in the FUS (left of gene-box) and TPL (right of gene-box). Color key represents the log2 (fold-change) of expression in AD compared to normal controls.

(f) PathView plot shows the cytokine-cytokine receptor interaction pathway (KEGG: hsa04060) and relevant genes involved in the HPC (left of gene-box) and PFC (right of gene-box). Color key represents the log2 (fold-change) of expression in AD compared to normal controls.

Figure S2.

(a-b) Venn diagrams show the overlap of DE genes in PSEN1-N/WT-N (a) or APOE4-N/WT-N (b) at a cutoff of FC>2 with AD-FUS/Normal at cutoffs of both FC>1.5 and P-value <0.05.

(c) Venn diagrams illustrate the overlap of DE genes between PSEN1-N, PSEN2-N and APOE4-N versus WT.

(d) GO enrichment analysis of the 1,372 common DE genes between PSEN2-N/WT-N and AD/Normal in the FUS.

(e) PathView plot shows part of the PI3K-AKT signaling pathway (KEGG: hsa04151) and relevant genes involved in iPSC-derived neurons (left of gene-box) and the FUS (right of gene-box). Color key represents log2 (fold-change) of expression in AD compared to normal controls.

Figure S3.

Protein-protein interactions (PPI) between known AD risk factors and 566 shared DE genes in PSEN1-N and AD-FUS. Blue dots represent known AD risk factors, while red and green dots stand for up- and down-regulated DE genes in AD, respectively.

Figure S4.

Protein-protein interactions (PPI) between known AD risk factors and 566 shared DE genes in APOE4-N and AD-FUS. Blue dots represent known AD risk factors, while red and green dots stand for up- and down-regulated DE genes in AD, respectively.

Figure S5.

(a-b) Boxplots show 5mC/5hmC/5fC/caC levels on the promoters (a) and gene bodies (b) of all Refseq genes in PSEN2-N and WT-N (all p-values <2.2e-16).

(c-d) Boxplots show 5mC/5hmC/5fC/caC levels on the promoters (c) and gene bodies (d) of all Refseq genes in PSEN1-N and WT-N (all p-values <2.2e-16).

(e-f) Boxplots show 5mC/5hmC/5fC/caC levels on the promoters (e) and gene bodies (f) of all Refseq genes in APOE4-N and WT-N (all p-values <2.2e-16).

(g) Boxplots show the differences in expression (FPKM) fold-changes in methylation-gain and loss on the gene bodies of all Refseq genes. P-values were calculated by Wilcoxon rank sum test (p<0.05 (*); p<0.01 (**); p<0.001 (***)).

Figure S6.

(a-b) Heatmaps show expression fold-changes and methylation fold-changes in PSEN1/WT (a) and APOE4/WT (b). The methylation data are from GSE79144 (n=44). FC: fold-change.

Supplementary Figure 1

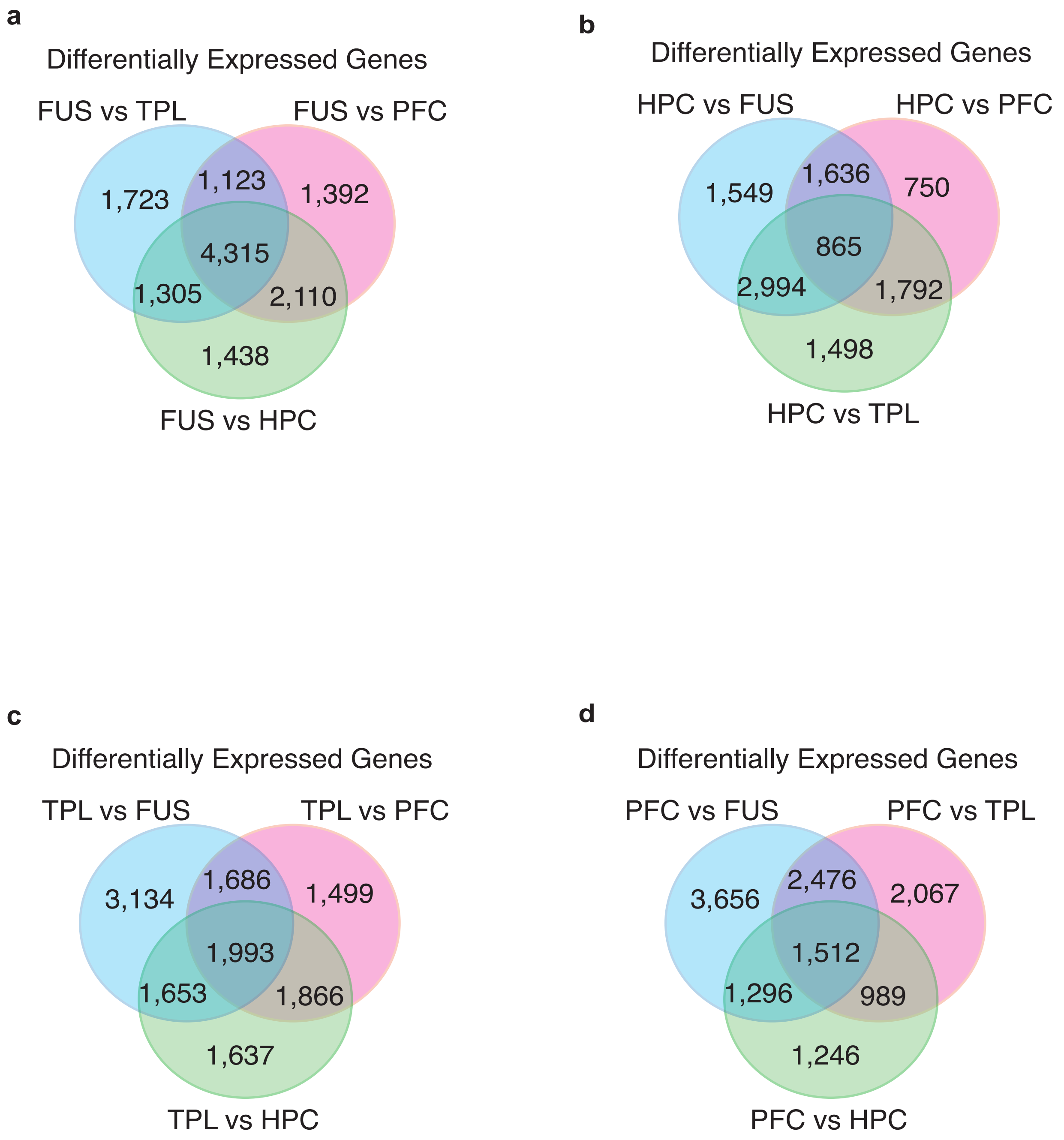

Supplementary Figure 1

e

Neuroactive Ligand-receptor Interaction

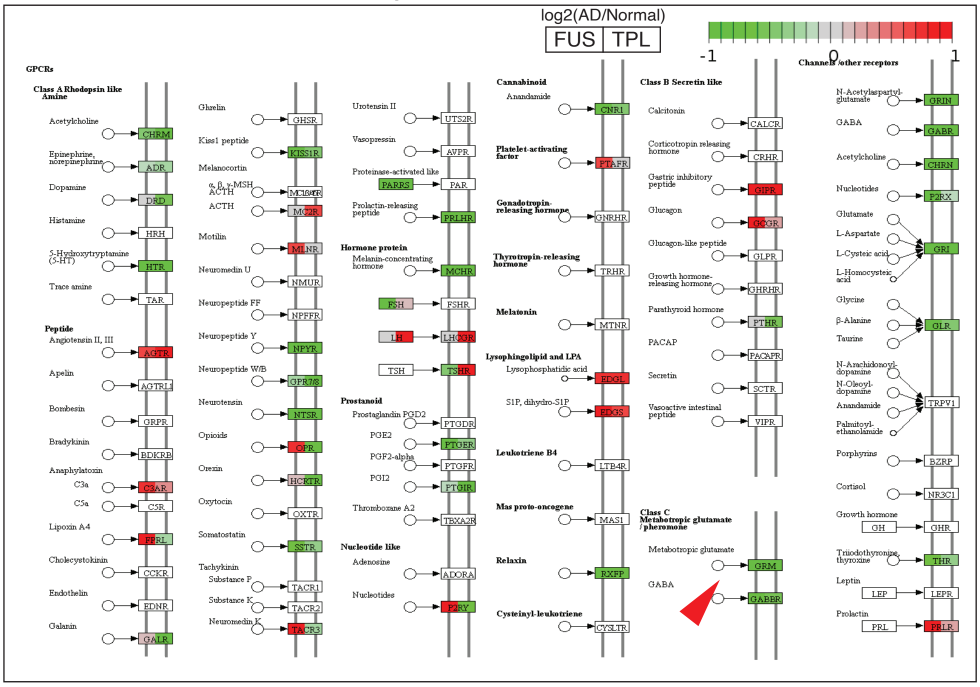

f

Cytokine-Cytokine Receptor Interaction

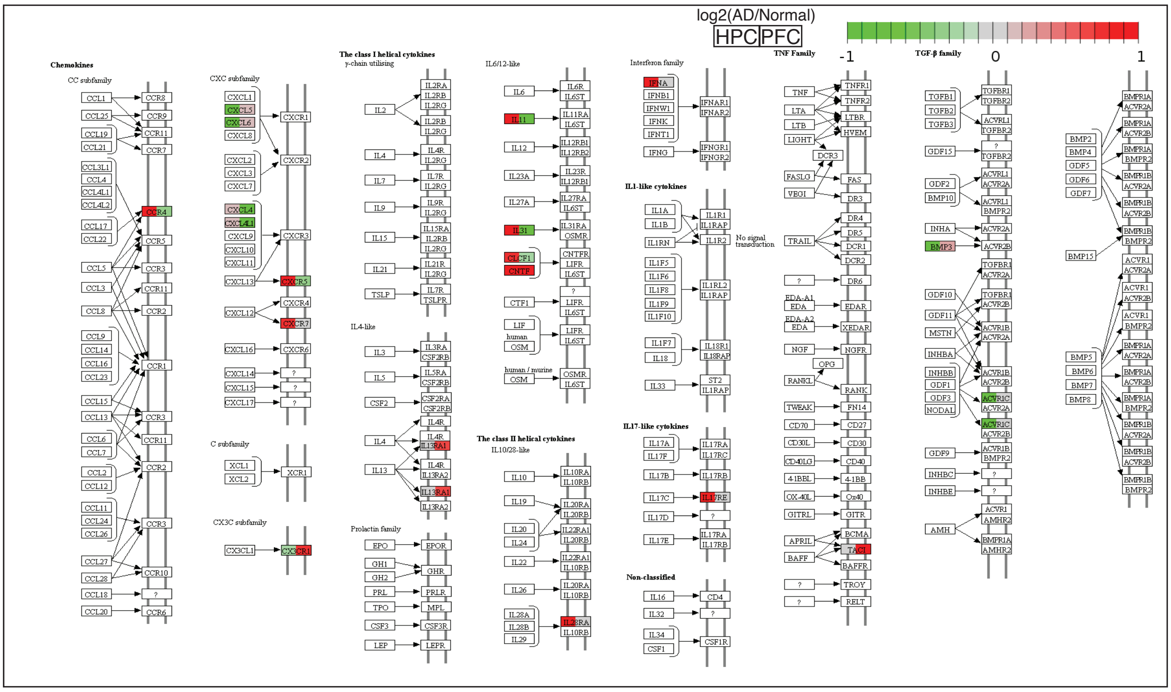

Supplementary Figure 2

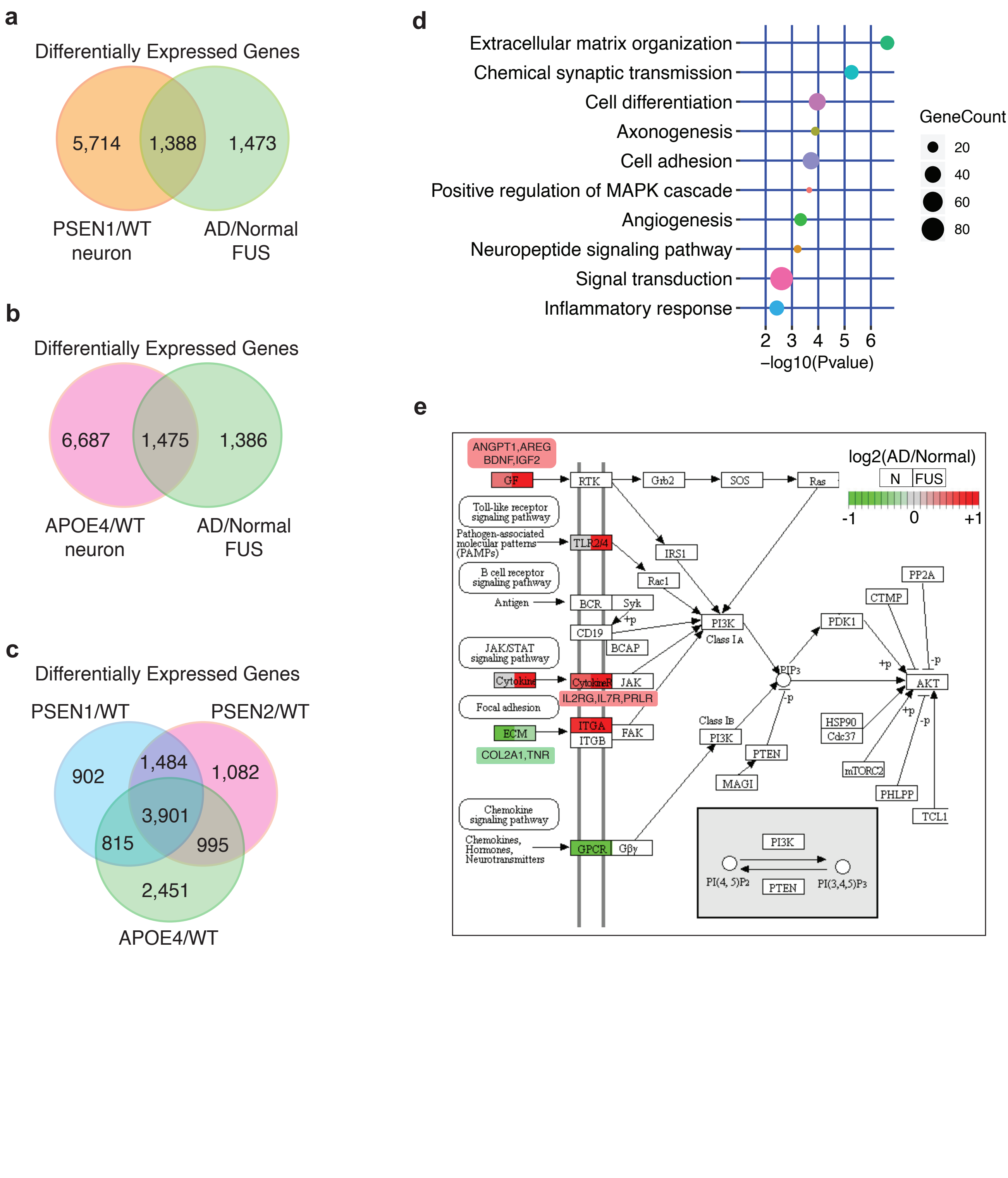

Supplementary Figure 3

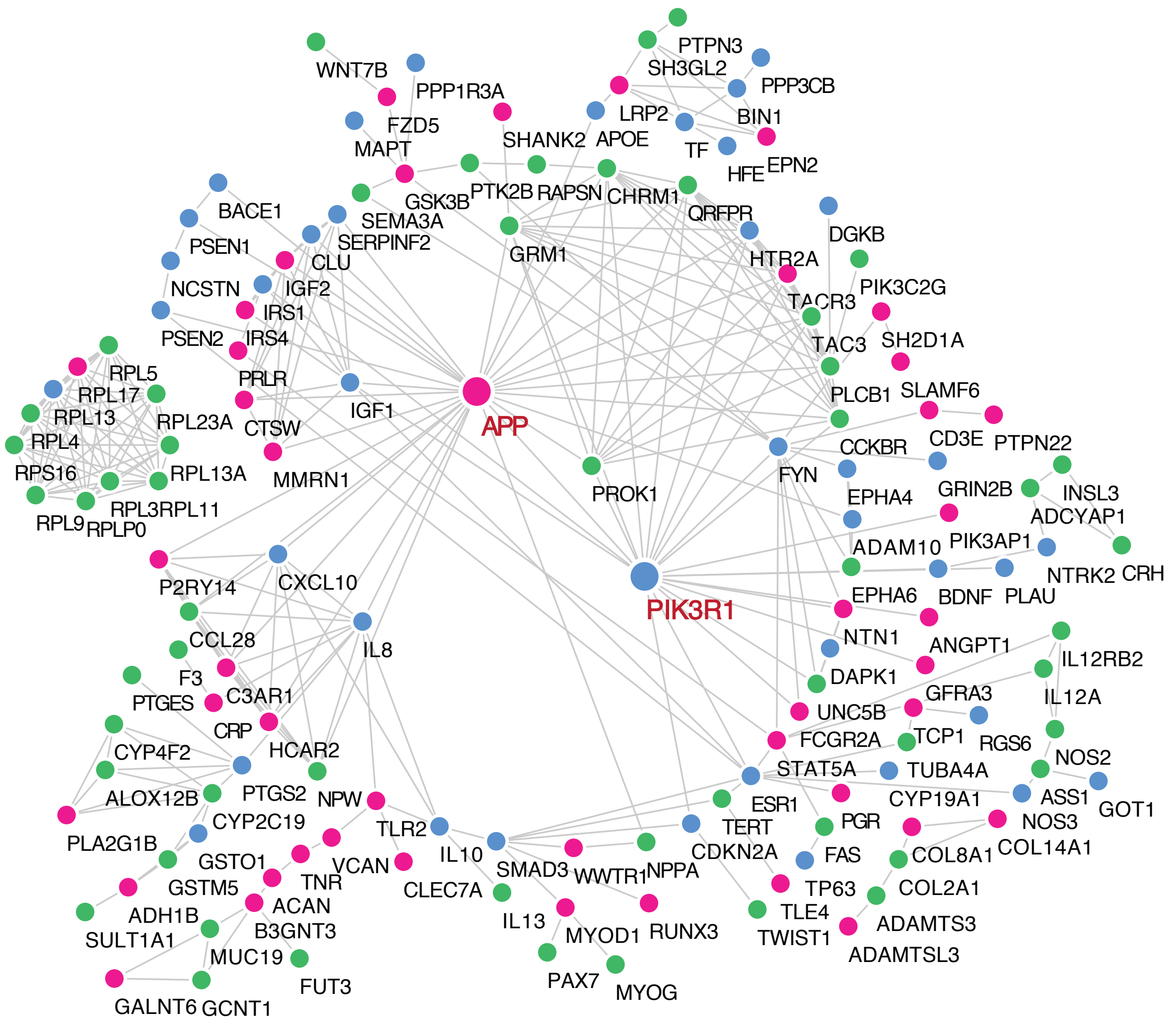

Supplementary Figure 4

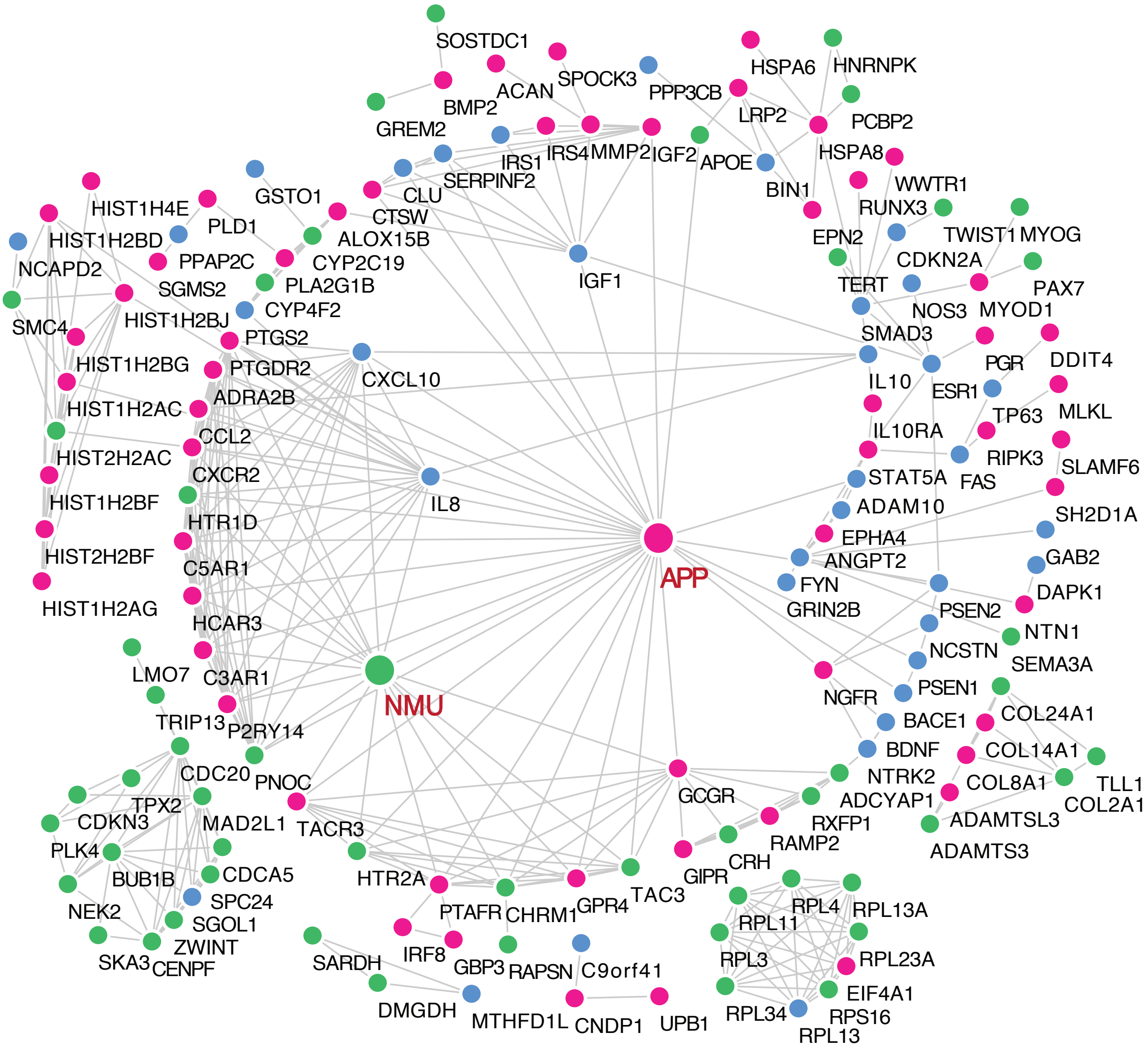

Supplementary Figure 5

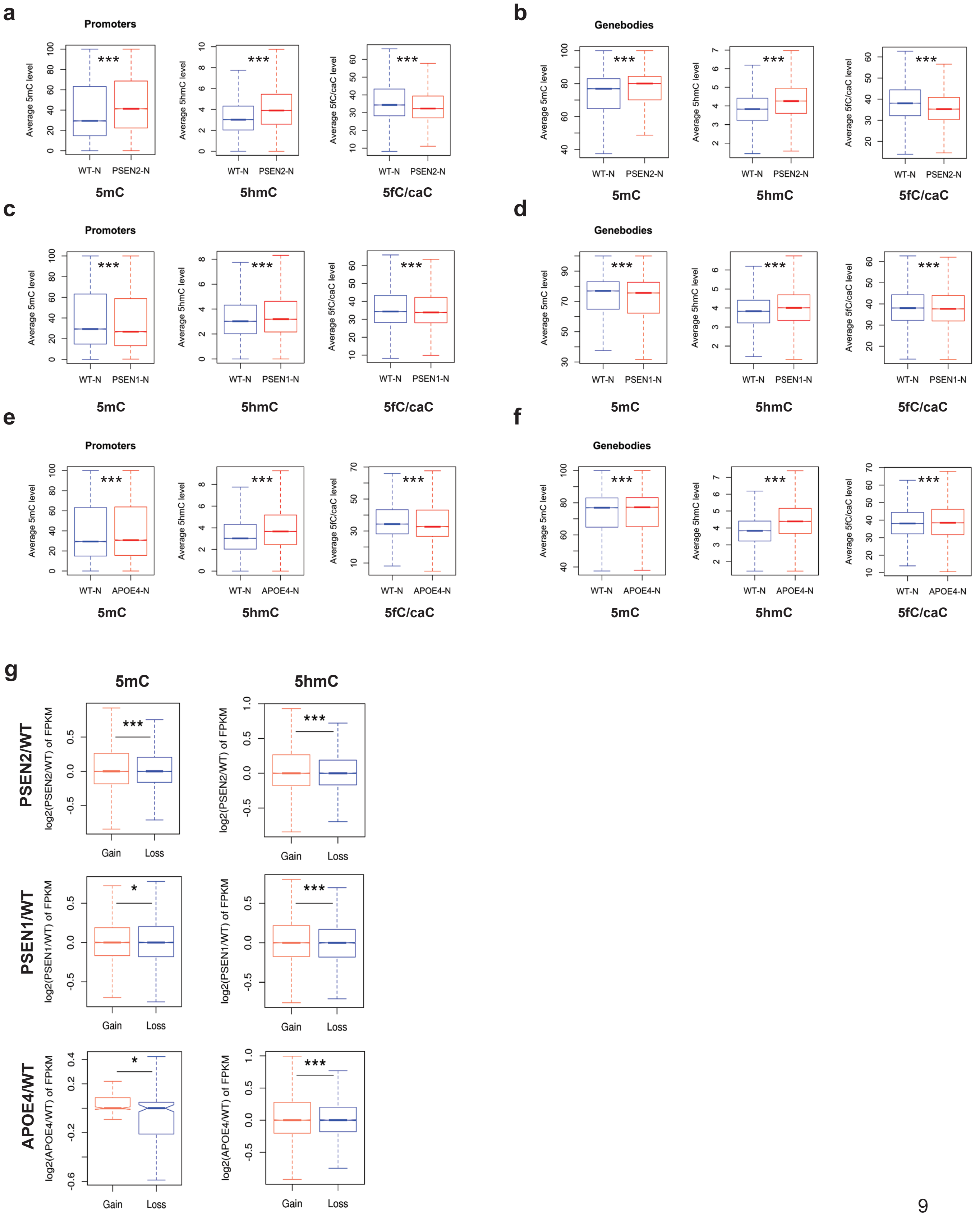

Supplementary Figure 6

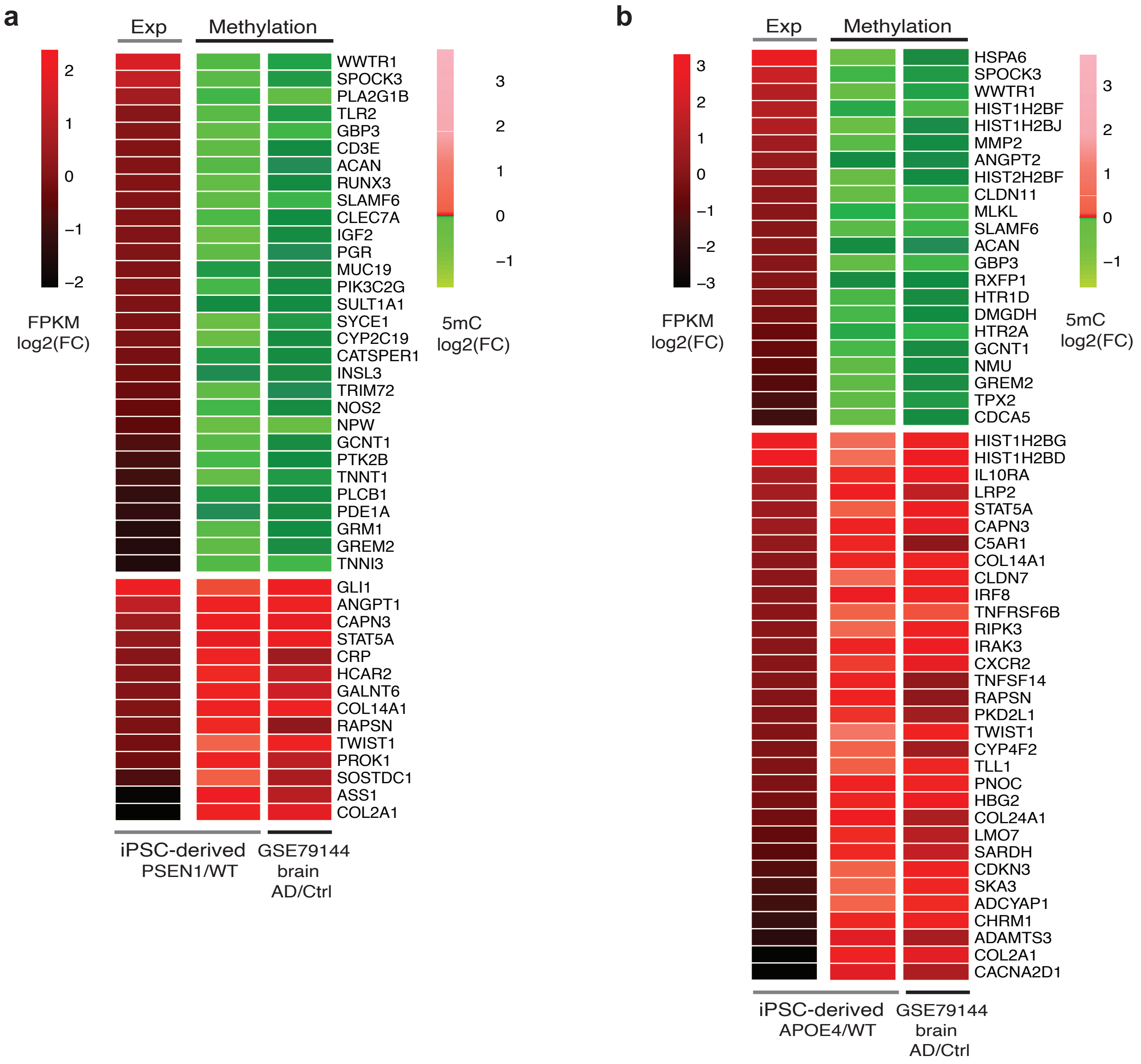

Supplement: Supplementary file 1 — Additional file 1: Figures S1-S6. Supplementary_figures_merge.pdf. [file 13148_2020_916_MOESM1_ESM.pdf]
